# Single-detector double-beam modulation for high-sensitivity infrared spectroscopy

**DOI:** 10.1038/s41598-023-44740-0

**Published:** 2023-10-25

**Authors:** Seong-Min Kim, Yow-Ren Chang, Young Jong Lee

**Affiliations:** Biosystems and Biomaterials Division, National Institute of Standards and Technology, Gaithersburg, MD 20899 USA

**Keywords:** Infrared spectroscopy, Infrared spectroscopy, Infrared spectroscopy

## Abstract

Balanced detection based on double beams is widely used to reduce common-mode noises, such as laser intensity fluctuation and irregular wavelength scanning, in absorption spectroscopy. However, employing an additional detector can increase the total system noise due to added non-negligible thermal noise of the detector, particularly with mid-infrared (IR) detectors. Herein, we demonstrate a new optical method based on double-beam modulation (DBM) that uses a single-element detector but keeps the advantage of double-beam balanced detection. The sample and reference path beams were modulated out-of-phase with each other at a high frequency, and their average and difference signals were measured by two lock-in amplifiers and converted into absorbance. DBM was coupled with our previously reported solvent absorption compensation (SAC) method to eliminate the IR absorption contribution of water in aqueous solutions. The DBM-SAC method enabled us to acquire IR absorption spectra of bovine serum albumin solutions down to 0.02 mg/mL. We investigated the noise characteristics of DBM measurements when the wavelength was either fixed or scanned. The results demonstrate that DBM can lower the limit of detection by ten times compared to the non-modulation method.

## Introduction

Infrared (IR) absorption spectroscopy has been widely used to characterize complex biomolecules, including proteins, nucleic acids, lipids, and carbohydrates^1^. The linear dependence of absorbance on concentration (Beer–Lambert law) enables quantitative concentration measurements, and detailed peak-shape analysis yields insights into the higher-order structures and configurations of biomolecules^2–4^. In particular, the amide I band of proteins is very sensitive to the secondary structures (e.g., α-helices, β-sheets, and random coils)^5,6^; so, it has been widely studied to not only identify proteins but also investigate the time-dependent protein denaturation^7–10^ and aggregation^11^. Furthermore, SI (international system of units)-traceability can significantly improve data reproducibility and inter-laboratory comparability for measurement assurance in biosciences and biotechnology. One of the most significant challenges for IR spectroscopies of biological samples is the strong IR absorption by water^12^.The substantial light absorption by the solvent reduces the transmitted signal intensity, dominates the dynamic range of a detection system, and overwhelms the analyte absorption contributions to the detected signal. These limitations have led to a thrust for more intense sources of IR light, such as synchrotron radiation^13–15^ and the more accessible external-cavity quantum cascade laser (EC-QCL)^16,17^.

The use of EC-QCLs was a significant step toward improving sensitivity in IR absorption measurements. EC-QCLs provide tunable, discrete frequency light ranging from mid- to far-IR wavelengths^16,17^ and have been extensively deployed for IR microscopy^18,19^ and spectroscopy^20,21^. The high intensity afforded by EC-QCLs significantly lowers the detection limit to < 1 mg/mL for the amide I band of proteins^22,23^. Alternatively, microfluidic modulation spectroscopy improved the signal-to-noise ratio (SNR) by measuring the signal difference as the sample and the solvent fluids were alternated in a microfluidic transmission cell^24^. To further improve system sensitivity, double-beam balanced detection schemes have been demonstrated by splitting an IR beam into a sample and reference beam and employing balanced detection. Lendl et al. reported IR spectra of proteins at concentrations as low as 0.1 mg/mL over the spectral range of 1500–1700 cm^−1^ using two-detector double-beam balanced detection^25^. More recently, we demonstrated that a new optical method, solvent absorption compensation (SAC), improved the sensitivity by greater than 100 times compared to the previous configuration without SAC, demonstrating a detection limit close to 0.2 mg/mL for the amide I band of protein^26^.

These recent advances in high-sensitivity IR spectroscopy are based on two-detector double-beam detection schemes either with subtraction^25^ or with division^26^. The double-beam balanced detection showed clear advantages in suppressing common-mode fluctuations, such as laser intensity and system temperature. Unfortunately, most mid- and far-IR detectors suffer substantial thermal noise even after being cooled thermo-electrically or by liquid nitrogen^27^, and employing an additional IR detector is accompanied by increased total system noise, and thus limiting the improvement of the system sensitivity.

In this work, we propose an optical method of double-beam balanced detection using a single detector. This new method is based on an out-of-phase intensity modulation of two beams and the simultaneous detection of modulated and unmodulated signals. We investigate the noise performance of this new double-beam modulation (DBM) method and compare it to double-detector measurements.

## Experimental section

### Optical configurations of double-beam modulation

**Figure 1:**
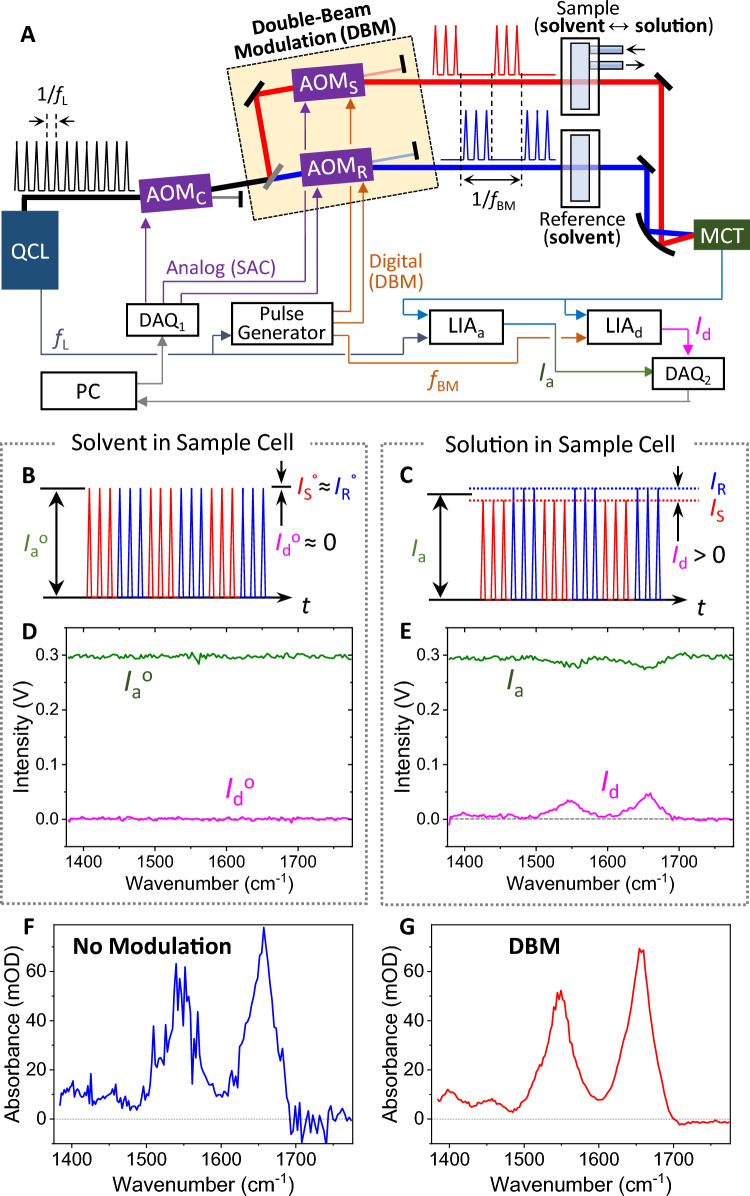
(**A**) Schematic diagram of the double-beam modulation (DBM) setup based on solvent absorption compensation (SAC) IR spectroscopy. See the text for details. (**B**, **C**) Illustration of time-dependent light intensity arriving on the MCT. The average intensity and the difference intensity are denoted as *I*_a_^o^ (*I*_a_) and *I*_d_^o^ (*I*_d_), respectively, when the sample cell is filled with (**B**) a solvent and (**C**) an analyte solution. *I*_S_^o^ (*I*_S_) and *I*_R_^o^ (*I*_R_) indicate the intensity through the sample path and the reference path, respectively. (**D**) Spectra of *I*_a_^o^ and *I*_d_^o^ for neat water. (**E**) Spectra of *I*_a_ and *I*_d_ for a BSA solution of 10 mg/mL. (**F**) Absorption spectrum calculated with no-modulation signals only (*I*_a_^o^ and *I*_a_) from Eq. (3). (**G**) Absorption spectrum by the DBM method, calculated with both average (*I*_a_^o^ and *I*_a_) and difference signals (*I*_d_^o^ and *I*_d_) from Eq. (2). The acquisition time for a single scan was 8 s.

Figure 1A shows the optical configuration of the one-detector DBM system used for this study. The system was based on the previously demonstrated solvent absorption compensation (SAC) IR spectroscopy^26^. An EC-QCL (MIRcat, DRS Daylight Solutions) tunable from 1376 to 1776 cm^−1^ and pulsed at 100 kHz (*f*_L_) with a duty cycle of 9.8% was used as a mid-IR source. Three acousto-optic modulators (AOM, Brimose) controlled the light intensity as a function of wavelength (for SAC) and modulated the two beams on and off (for DBM). The beam was first diffracted by an AOM unit (denoted as AOM_C_), which compensated for the strong light absorption of solvent by attenuating the light intensity as a function of wavelength. The beam from AOM_C_, was then split into sample and reference paths and passed through another AOM, denoted as AOM_S_ and AOM_R_ for the sample and reference paths, respectively. AOM_S_ and AOM_R_ control both the intensity and on-off phase of the beams. The beams from AOM_S_ and AOM_R_ diffracted in the opposite direction to the first diffraction direction at AOM_C_ for compensation of wavenumber-dependent dispersion. After passing through a sample cell and a reference cell separately, the two parallel beams were focused by an off-axis parabolic mirror (effective focal length = 75 mm, MPD239-M03, Thorlabs) onto a single-element thermo-electrically cooled MCT detector (MCT-7-TE4, Infrared Associates).

### Electronic control and signal processing

Each AOM was controlled by a radio-frequency (RF) driver that had two inputs: an analog port and a digital port. Analog signals were generated by a current output device (NI9265, National Instruments, denoted as DAQ_1_). Digital pulses generated by a pulse generator (DG535, Stanford Research Systems) modulated the two beam's on-off out-of-phase to each other at a desired beam modulation frequency (*f*_BM_). The signal from the single MCT detector was processed by two lock-in amplifiers (LIA, SR830, Stanford Research System) in parallel. One LIA (denoted as LIA_a_) measured the average signal (*I*_a_) synchronized with *f*_L_ received from the EC-QCL driver. The other LIA (denoted as LIA_d_), synchronized with *f*_BM_, measured the difference signal (*I*_d_). *I*_a_ and *I*_d_ signals were simultaneously read by a multifunctional I/O device (PCIe-6374, National Instruments, denoted as DAQ_2_) attached to a control/acquisition computer. The time constant for both LIAs was 30 ms for spectral scanning measurement and 10 ms for fixed-wavelength measurement. The sensitivities were 1 and 0.2 V for LIA_a_ and LIA_d_, respectively.

The analog voltage sequences for the three AOMs were pre-calibrated with a solvent in both sample and reference cells so that *I*_a_ = constant (for SAC) and *I*_d_ = 0 (for DBM) during spectral scanning. The laser wavelength was scanned in a continuous sweep mode from 7.26 μm (1377 cm^−1^) to 5.63 μm (1776 cm^−1^) with a scanning speed of 0.2 μm/sec, which corresponded to 8 s for a single scan. During scanning, the laser system generated 0.5 ms-width pulses every 0.01 μm. The wavelength trigger pulses were synchronized to DAQ_1_, allowing the AOMs to adjust the diffraction efficiencies according to the calibrated voltage sequences. The wavelength trigger pulses were also connected to DAQ_2_ to locate the exact wavelength, while *I*_a_ and *I*_d_ were recorded.

### Sample flow cell and sample preparation

The sample cell and the reference cell were liquid flow cells (GS20572, Specac) consisting of two 3 mm thick CaF_2_ windows and a lead spacer with a nominal path length of 26 μm. The reference cell was filled with distilled water and sealed during the measurement. The sample cell was filled with either an analyte solution or a solvent. A syringe pump (Pump 33 DDS, Harvard Apparatus) was used to replace liquids in the sample cell. A volume of 600 µL liquid was infused with a flow rate of 50 µL/min, and a single liquid replacement took 12 min. The entire optical system was enclosed and continuously purged with dry air. Bovine serum albumin (BSA, > 96%, Sigma-Aldrich) was used as received. A stock solution of BSA in distilled water was prepared at 10 mg/mL and diluted to desired concentrations in the range of 1 down to 0.02 mg/mL.

## Results and discussion

### One-detector double-beam modulation (DBM)

Figure 1A shows a schematic diagram of the DBM-SAC IR spectroscopy system. The three AOMs modulated the two beams out-of-phase to each other and simultaneously adjusted their intensities for SAC. Figure 1B, C shows simplified illustrations of modulated light pulses detected by a single detector. The red pulses indicate light, *I*_S_, through the sample path, while the blue pulses indicate light, *I*_R_, through the reference path. When the sample cell was filled with the solvent, the intensity difference between *I*_S_ and *I*_R_ was set to zero. When analytes in the sample cell absorbed the light, the intensity difference between *I*_S_ and *I*_R_ became nonzero. The average signal, *I*_a_ = (*I*_S_ + *I*_R_)/2, was measured with a lock-in amplifier (LIA_a_) synchronized with the laser pulse frequency, *f*_L_, with a time constant sufficiently longer than the beam modulation period. The difference signal, *I*_d_ = *I*_S_ – *I*_R_, was measured by another lock-in amplifier (LIA_d_) synchronized with the beam-modulation frequency, *f*_BM_. Figure 1D shows the spectra of *I*_a_ and *I*_d_ for the solvent. *I*_a_ and *I*_d_ were adjusted to be constant by the solvent absorption compensation technique, which used the full dynamic range of the detection system and reduced the dynamic range associated noise contribution. In the presence of absorbing analytes in the sample cell, *I*_a_ decreased, and *I*_d_ increased at absorption frequencies, shown in Fig. 1E.

The observed *I*_a_ and *I*_d_ signals can be converted to absorbance by a simple expression. The intensities of *I*_S_ and *I*_R_ are

1 $$\left\{ { \begin{array}{*{20}c} {I_{{\text{S}}} = I_{{\text{a}}} - \frac{1}{2} I_{{\text{d}}} } \\ {I_{{\text{R}}} = I_{{\text{a}}} + \frac{1}{2} I_{{\text{d}}} } \\ \end{array} } \right.$$

Then, DBM absorbance, *A*_DBM_ can be expressed as

2 $$A_{{{\text{DBM}}}} = - \log \left( {\frac{{I_{{\text{S}}} /I_{{\text{R}}} }}{{I_{{\text{S}}}^{{ {\text{o}}}} /I_{{\text{R}}}^{{\text{ o}}} }}} \right) = - \log \left[ {\frac{{\left( {2I_{{\text{a}}} - I_{{\text{d}}} } \right)}}{{\left( {2I_{{\text{a}}} + I_{{\text{d}}} } \right)}}} \right] + \log \left[ {\frac{{\left( {2I_{{\text{a}}}^{{ {\text{o}}}} - I_{{\text{d}}}^{{ {\text{o}}}} } \right)}}{{\left( {2I_{{\text{a}}}^{{ {\text{o}}}} + I_{{\text{d}}}^{{\text{ o}}} } \right)}}} \right]$$

where *I*_S_^o^ and *I*_R_^o^ are intensities when a solvent is in the sample cell. Even though the system used a single detector, the DBM technique still took advantage of a double-beam configuration, which suppressed common-mode fluctuations between the two beam paths using their intensity ratio, (*I*_S_/*I*_R_)/(*I*_S_^o^/*I*_R_^o^). To examine the advantage of a double-beam configuration, we compare the DBM absorbance with a no-modulation absorbance. The no-modulation absorbance, *A*_no-mod_, was calculated only with *I*_a_^o^ and *I*_a_ without considering the modulation signals, *I*_d_^o^ and *I*_d_. For this calculation, we assumed that the reference path signals were equal for *I*_R_ = *I*_R_^o^ = *I*_S_^o^, which led to *I*_d_^o^ = 0 and *I*_d_ = 2(*I*_a_^o^ − *I*_a_) in Eq. (2). Then, *A*_no-mod_ was expressed as

3 $$A_{{{\text{no}} - {\text{mod}}}} = - \log \left[ {\left( {2I_{{\text{a}}} - I_{{\text{a}}}^{{\text{o}}} } \right)/I_{{\text{a}}}^{{\text{o}}} } \right]$$

Figure 1F, G shows the two absorption spectra of *A*_DBM_ and *A*_no-mod_ generated from the identical signal set of *I*_a_^o^, *I*_d_^o^, *I*_a_, and *I*_d_. Unambiguously, the *A*_DBM_ spectrum in Fig. 1G shows a significantly higher SNR than the *A*_no-mod_ spectrum in Fig. 1F. The newly proposed DBM method considered both the average signals and the difference signals between the double paths to effectively suppress the common-mode noise, leading to the demonstrated improved SNR with a single detector.

### Noise comparison of one-detector DBM and other conventional methods

**Figure 2:**
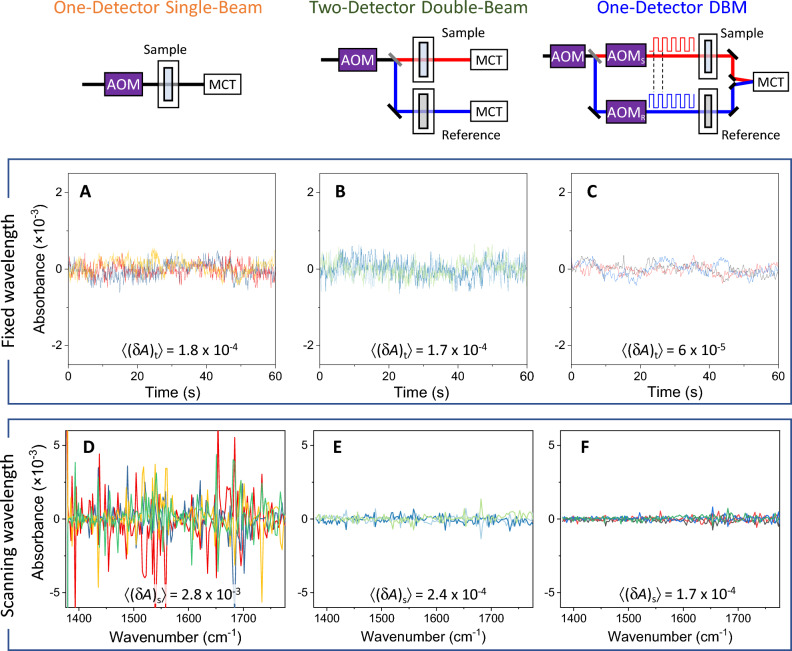
Comparison of three absorption measurement schemes and their temporal and spectral noises. (**A**–**C**) Absorbance signals measured at a fixed wavelength by using the mean value as the reference for the absorbance calculation for 1 min with a sampling period of 0.1 s. The standard deviation of each trace was used to represent the temporal noise, denoted as (δ*A*)_t_, and 〈(δ*A*)_t_〉 indicates the mean of three (δ*A*)_t_ values. The laser frequency was fixed at 1376 cm^−1^. (**D**–**E**) Absorbance measured from water while laser wavelength was scanned. Three spectra were measured consecutively in each scheme. The mean of the three spectra was used as the reference spectrum for absorbance calculation. The standard deviation of the absorbance in each spectrum was used to represent the spectral noise, denoted as (δ*A*)_s_. The displayed values 〈(δ*A*)_s_〉 at the bottom of the panels are the mean of the three (δ*A*)_s_ values. The LIA time constants were 10 ms, and the sampling time was 0.1 s. The frequency of beam modulation, *f*_BM_, was 50 kHz for the one-detector DBM scheme.

Figure 2 shows schematics of two conventional absorption measurement methods (one-detector single-beam and two-detector double-beam) compared with the newly proposed one-detector DBM method. First, we examined the temporal noise characteristics at a fixed wavelength. For each scheme, transmitted light intensity was monitored for one minute with a sampling period of 0.1 s and converted to absorbance by using the mean intensity as the reference intensity. Figure 2A–C show three sequences of temporal absorbance fluctuations for each detection method. The standard deviation of the time profile of each profile is denoted as (δ*A*)_t_, and the mean of three profiles is denoted as 〈(δ*A*)_t_〉, which are displayed in each panel of Fig. 2A–C. It is noted that 〈(δ*A*)_t_〉 measured by the one-detector single-beam method is similar to 〈(δ*A*)_t_〉 by the two-detector double-beam method. Given that the one-detector single-beam method accompanies the common-mode noise while the two-detector double-beam method contains additional detector noise, it suggests the common-mode noise reduction at a fixed wavelength is analogous to the additional detector noise. We also note that 〈(δ*A*)_t_〉 by the one-detector DBM method is three times smaller than the other two values, which suggests the reduced detector noise is significant.

Figure 2D–E show spectral noises measured with the three absorption measurement methods using water both for sample and reference. The standard deviation of absorbance over the frequency steps is used to represent the spectral noise, denoted as (δ*A*)_s_. The mean value, 〈(δ*A*)_s_〉, from three consecutively measurements is displayed in each panel of Fig. 2D–E. First, 〈(δ*A*)_s_〉 measured while the wavelength was scanned (Fig. 2D) is significantly larger than 〈(δ*A*)_t_〉 measured at a fixed wavelength (Fig. 2A), suggesting that wavelength scanning-related fluctuations were significant. Also, it is noted that 〈(δ*A*)_s_〉 measured by the one-detector single-beam method is more than ten times larger than 〈(δ*A*)_s_〉 of the other double-beam methods. This suggests that the scanning-related fluctuations occurred before beam-splitting, and the common-mode noises were greatly reduced by the two double-beam methods. Furthermore, between the two double-beam methods, the one-detector DBM method of Fig. 2F showed a smaller 〈(δ*A*)_s_〉 than the two-detector method of Fig. 2E. This suggests that the additional detector noise was non-negligible in the conventional two-detector double-beam method and was reduced by using the single-detector DBM. The results shown in Fig. 2 demonstrated that the new one-detector DBM method reduced spectral scanning noise significantly by taking advantage of both double-beam common-mode compensation and single-element detection.

Table 1 summarizes probable noise sources associated with the three absorption measurement methods and visualizes their contributions to the observed noises displayed in Fig. 2. Because the SAC method was used for all three methods, the difference in the dynamic range noise contribution was minimized among the methods. Fluctuations in laser intensity and humidity can be considered common-mode noises that can occur whether the wavelength is scanned or not. Scanning-associated noises can be due to the mismatch between the acquired wavelengths defined by wavelength trigger pulses from the laser controller and the actual wavelengths, the beam pointing instability of the laser during spectral scanning or the AOM efficiency fluctuations. The scanning-related noises are common-mode noises and can be effectively compensated by a double-beam measurement scheme.

**Table 1:** Schematic comparison of noise sources and their contribution to observed temporal (fixed-wavelength) noises and spectral (scanning-wavelength) noises.

Noise source		Relation	One-detector single-beam	Two-detector double-beam	One-detector DBM
Common-mode	Fixed-wavelength (intensity, humidity)	*a*	*	·	·
	Scanning-wavelength (λ mismatch, AOM)	*b*	*******	*	*
Detector (MCT, pre-amplifier)		*c*	*	**	*
Temporal noise, (δ*A*)_t_ (Fig. 2A–C)		(*a* + *c*)	**	**	*
Spectral noise, (δ*A*)_s_ (Fig. 2D–F)		(*a* + *b* + *c*)	*********	***	**

The noise level of each noise source is represented by the number of the symbol (*). The observed noises at the two bottom rows are represented in the linear sum of contributing noise levels for visualization although the actual noise propagations are more complex.

### Optimization of acquisition parameters for DBM

**Figure 3:**
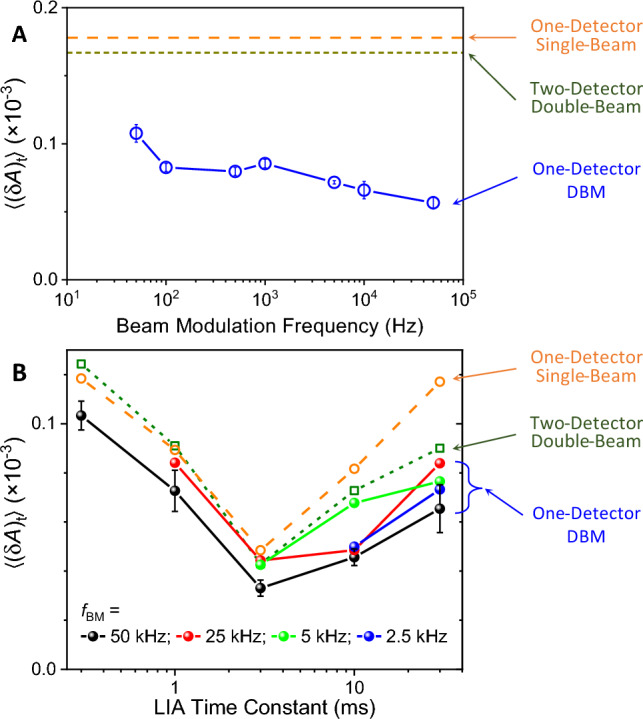
(**A**) Plot of temporal noise average, 〈(δ*A*)_t_〉, as a function of the beam modulation frequency (*f*_BM_) measured by the DBM method. For the data of (A), absorbance signals was monitored for 1 min with a sampling period of 0.1 s. 〈(δ*A*)_t_〉 was calculated in the same was of Fig. 2A–C. (**B**) Plots of 〈(δ*A*)_t_〉 as a function of the LIA time constant with various *f*_BM_. The DBM results are displayed as the filled spheres, while the results of the other methods are displayed as the empty circles and squares. For the data of (B), absorbance was monitored for 1 min with a sampling period of 0.3 s. For comparison, the dashed orange lines and the dotted green lines are plotted to represent the one-detector single-beam and two-detector double-beam methods, respectively.

**Figure 4:**
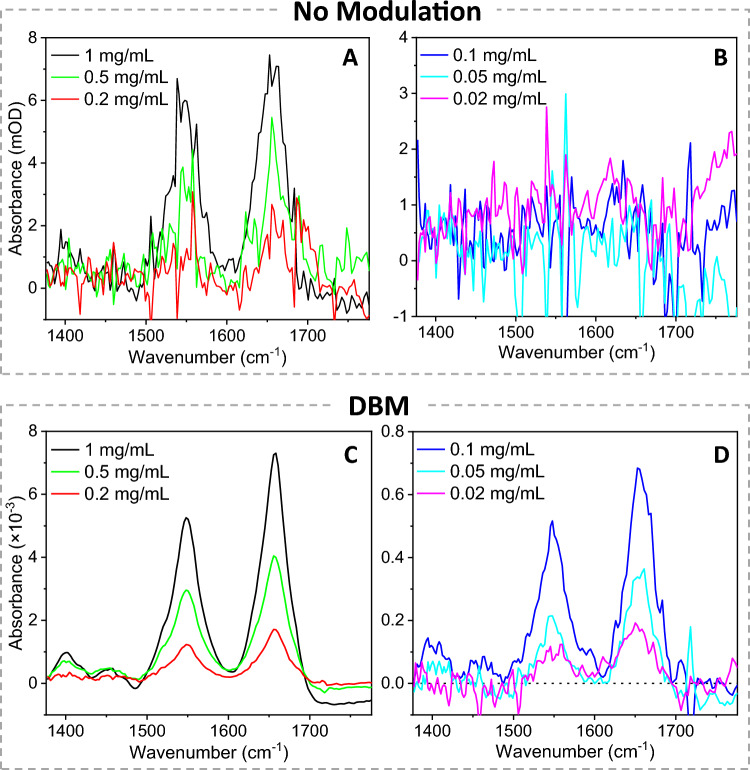
Comparison of absorption spectra measured by the DBM and the non-DBM methods for various BSA concentrations. (**A**, **B**) Absorption spectrum calculated with no-modulation signals only (*I*_a_^o^ and *I*_a_) from Eq. (3). (**C**, **D**) Absorption spectrum by the DBM method, calculated with both average (*I*_a_^o^ and *I*_a_) and difference signals (*I*_d_^o^ and *I*_d_) from Eq. (2). The spectra in (**B**, **D**) were vertically translated to match absorbance as zero at 1695 cm^−1^. Twenty wavelength scans were measured for a BSA solution in the sample cell. Then, the solution was replaced with water, and another twenty wavelength scans were measured. The net signal acquisition time was 12 min per solution-solvent pair, excluding time for exchanging liquids. The displayed plots were the average spectra of three solution-solvent exchange cycles.

We examined the effects of a few acquisition parameters on DBM measurement. Figure 3A shows (δ*A*)_t_ measured with several beam modulation frequencies (*f*_BM_) employed for the DBM method. Overall, 〈(δ*A*)_t_〉 decreased monotonically as *f*_BM_ increased. The highest *f*_BM_ we tested was 50 kHz, meaning that every other pulse was on-off modulated from 100 kHz laser pulses. Another parameter we examined was the time constant (τ) of the two LIA. As a rule of thumb, a longer τ will reduce the noise but increase the acquisition time per data point. We examined the noise characteristics when τ varied. To keep the total acquisition time the same, we adjusted the number of averaged scans reciprocally to τ. In Fig. 3B, 〈(δ*A*)_t_〉 are plotted as a function of τ for all three measurement methods. For the DBM methods, 〈(δ*A*)_t_〉 are plotted for four different *f*_BM_. The raw time profiles are shown in Fig. S1. Interestingly, in all examined conditions, 〈(δ*A*)_t_〉 showed the minimum value at τ = 3 ms (Sampling period: Δ*t* = 15 ms). The non-monotonic τ-dependence of 〈(δ*A*)_t_〉 indicates that multiple noise sources with different characteristic time scales contribute to the temporal noise of the three detection methods to similar extents. It must be noted that the optimal τ for the minimum 〈(δ*A*)_t_〉 in Fig. 4B was based on the fixed-wavelength measurements. However, the actual spectral acquisition time was limited by various timing factors, such as a reliable wavelength-scanning speed and initialization time for each scan. From trials of multiple combinations of laser scanning and acquisition parameters, we found the optimal parameters of the currently employed spectroscopy system to be τ = 30 ms, Δ*t* = 100 ms, and speed = 0.2 μm/s, which were used for the following spectrum measurements of protein samples.

### Absorption spectra of BSA solutions by the DBM method

Using the optimized DBM method, we acquired absorption spectra of BSA solutions at various concentrations. First, we calculated the absorption spectra using only the non-modulation signals (*I*_a_ and *I*_a_^o^) with Eq. (3), and the spectra are shown in Fig. 4A, B. Using the SAC method, the absorption spectrum of a 0.2 mg/mL BSA solution showed the amide I peak with an SNR of ~ 3, similar to the earlier report of SAC-IR measurements of proteins.^26^ In contrast, Fig. 4C, D shows the absorption spectra calculated with Eq. (2) using both the average signals (*I*_a_ and *I*_a_^o^) and the beam modulation signals (*I*_d_ and *I*_d_^o^). Figure 4A, B and C, D were constructed from identical spectral data for each BSA solution, but the spectra calculated by the DBM method (Fig. 4C, D) showed greatly reduced spectral noise compared to those generated by non-modulation data only (Fig. 4A, B). The comparison between Fig. 4A and D indicates that the DBM method improved the SNR by greater than ten times compared to non-DBM measurements.

As the temporal and spectral noises are reduced by the DBM method, previously neglected noise sources become noticeable. For example, multiple liquid exchange of solution and solvent caused the offset of an absorption spectrum to be translated vertically. Figure S2A shows the original absorption spectra measured from low-concentration BSA solutions. The offset drift was compensated by simple vertical translation (see the corresponding spectra in Fig. 4D). We found that the vertical drift of baseline absorbance was in the range of 10^–4^, making the offset drift correction necessary for absorption measurement of protein concentration below 0.1 mg/mL.

**Figure 5:**
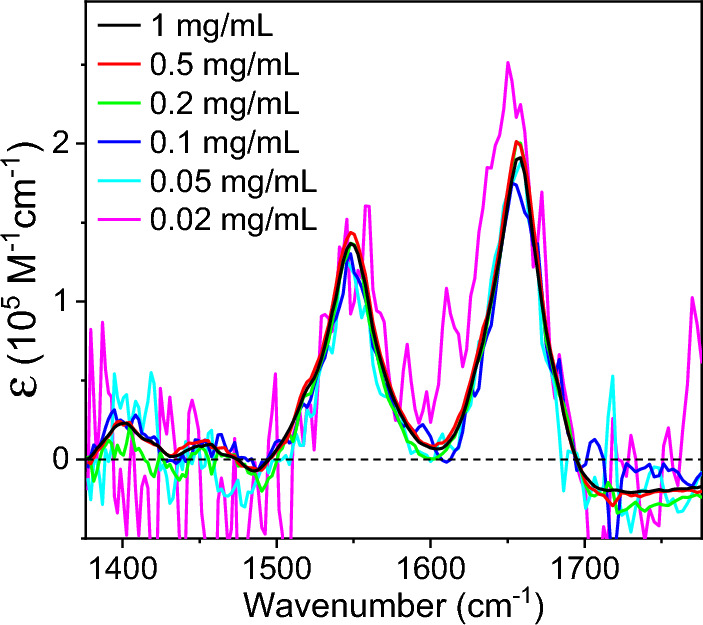
Molar absorption coefficient (*ε*) spectra calculated by scaling the absorbance spectra shown in Fig. 4C, D measured with BSA solutions at six different concentrations measured by the DBM method.

The Beer–Lambert law explains the linear dependence of absorbance on the analyte concentration as *A* = *εcl*, where *ε*, *c*, and *l* are absorption coefficient, concentration, and path length, respectively. We examined the concentration linearity of the absorption spectra acquired by the DBM method by comparing the concentration-scaled spectra. Figure 5 shows the *ε*-spectra calculated from the absorption spectra of BSA solutions at six different concentrations, where *ε* = *A*/(*cl*) and *l* = 26 μm. The *ε* spectra of the solutions from 1 mg/mL down to 0.02 mg/mL showed good agreement with each other, confirming the linear concentration dependence of absorbance measured by the DBM method.

### Limit of detection

The limit of detection (LOD) of a spectral measurement can be defined in a simple form as LOD = (3 × noise)/(signal slope). However, it should be noted that LOD can vary depending on the definition of the noise and the signal and the experimental conditions, particularly in molecular detection by multi-frequency spectroscopy. In this study, we define the signal in two ways: the absorbance at a single frequency and the mean absorbance over a frequency range. We examined the amide I and II bands to determine the LOD of BSA solution in water. The signal slopes of the single-frequency and the mean-band absorbances of the amide I mode were 7.3 × 10^–3^ (mg/mL)^−1^ and 2.9 × 10^–3^ (mg/mL)^−1^, respectively. For the amide II band, the slopes of single-frequency and mean-band absorbances were 5.3 × 10^–3^ (mg/mL)^−1^ and 2.3 × 10^–3^ (mg/mL)^−1^, respectively. The signal slopes were determined by linear fitting with the *y*-intercept of zero.

Similar to the signal, the noise cab be defined variously in the LOD definition depending on the experimental condition, the acquisition time, and any data post-processes. For example, if we use the temporal noise 〈(δ*A*)_t_〉 at a fixed wavelength, the noise will be 6 × 10^–5^, shown in Fig. 2C. If we use the spectral noise 〈(δ*A*)_s_〉 from Fig. 2F, the noise will become 1.7 × 10^–4^. We can also estimate the spectral noise observed from the absorbance spectrum of a low concentration BSA solution. Figure S3 shows that the noisy absorption spectrum of a 0.1 mg/mL BSA solution was subtracted by the high S/N spectrum of a 1 mg/mL solution after concentration-scaling, and the standard deviation of the absorbance residual was 4.0 × 10^–5^. We can also define the standard deviation of the signals (peak or mean absorbances) over multiple absorption spectra measurements performed via sample-solvent exchange. Then, the standard deviation of single-frequency absorbances were 8.2 × 10^–4^ and 5.6 × 10^–5^ for the amide I and amide II bands, respectively. Furthermore, the standard deviation of mean-band absorbances were 4.5 × 10^–6^ and 4.1 × 10^–6^ for the amide I and amide II bands, respectively, which are even lower than the single-frequency absorbance results.

As discussed above, both the signal slope and the noise can be defined differently, and thus, the LOD can also be differently determined. If an absorbance measurement is performed at a fixed wavelength tuned to the amide I band with a sample time of 0.1 s, the LOD will become 3 × (6 × 10^–5^)/(7.3 × 10^–3^ (mg/mL)^−1^) = 0.02 mg/mL. If a protein concentration is determined from an absorbance spectrum, which took 12 min per spectrum, the LOD of single-frequency absorbance at the amide I band will be 3 × (8.2 × 10^–5^)/(7.3 × 10^–3^ (mg/mL)^−1^) = 0.03 mg/mL. The mean-band absorbance over a spectral range can lower the LOD further down to 3 × (4.5 × 10^–6^)/(5.3 × 10^–3^ (mg/mL)^−1^) = 0.003 mg/mL.

**Figure 6:**
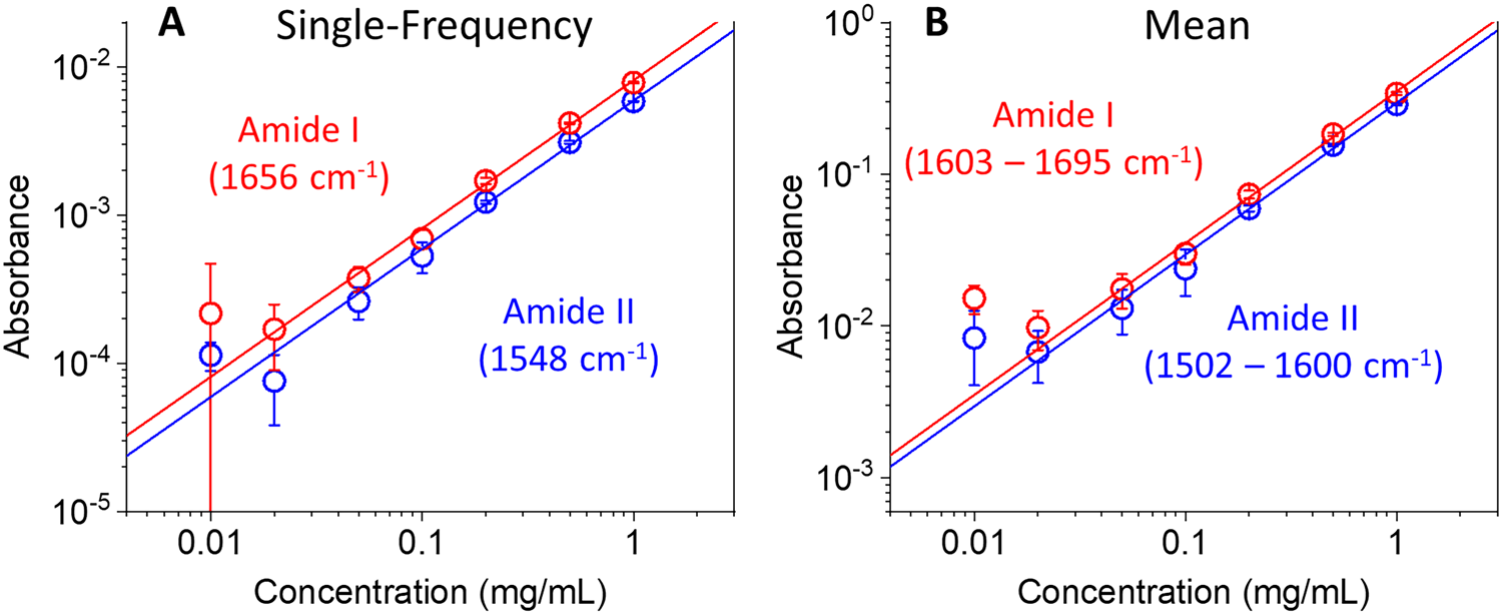
Log–log plots of (**A**) single-frequency absorbance and (**B**) mean-band absorbance as a function of BSA concentration. In all panels, the circles and the error bars represent the average absorbance and the standard deviation, respectively, of three repeated measurements; and the solid lines indicate the best linear fits with zero *y*-intercepts.

The above LOD estimations are based on the noise determined by signal reproducibility, not by the linearity of the signal to the analyte concentration. Figure 6 shows log–log scatter plots of absorbance as a function of BSA concentration. Both the peak absorbance and the mean-band absorbance were measured repeatedly for three solution-solvent exchange cycles. In Fig. 6A, the peak absorbance results showed an excellent linear concentration dependence from 1 mg/mL down to 0.02 mg/mL and deviated from the best fit line below 0.02 mg/mL, which is comparable to the LOD from spectral noise measurement. In Fig. 6B, the mean-band absorbance also showed the deviation from the linear fit below 0.02 mg/mL, which is far higher than the LOD from spectral noise (0.003 mg/mL). Thus, based on the above-described measurement conditions of the current liquid-exchange system, the LOD of mean-band absorbance of the BSA amide I peak can be said to be 0.02 mg/mL. It should be noted that this demonstrated LOD can vary depending on the measurement configuration, the signal type, the averaging period, and the liquid flow system performance. However, compared to our previous SAC-IR spectroscopy results, we find the LOD of the DBM-employed SAC-IR spectroscopy is lower by at least an order of magnitude.

## Conclusion

We have demonstrated that the DBM method uses a single-element detector but retains the advantage of double-beam balanced detection. The DBM method, coupled with the SAC method, enabled the acquisition of IR absorption spectra of BSA solutions down to 0.02 mg/mL. Comparisons of the noise characteristics between the new DBM method and two other conventional methods (one-detector single-beam and two-detector double-beam) showed that DBM could lower the limit of detection by ten times compared to the non-modulation methods. This new DBM method can be used to improve the sensitivity of other double-beam optical systems whose sensitivity may be limited by the detector noise.

### Supplementary Information

Supplementary Information.

## Data Availability

The datasets generated during and/or analyzed during the current study are available from the corresponding author upon reasonable request.
