# Supplementary material for: Single-detector double-beam modulation for high-sensitivity infrared spectroscopy
Source: Sci Rep. 2023 Oct 25;13:18231. doi: 10.1038/s41598-023-44740-0 (PMC10600177; doi:10.1038/s41598-023-44740-0)
Supplement: Supplementary file 1 — Supplementary Information. [file 41598_2023_44740_MOESM1_ESM.docx]

**Supplementary Information for**

Single-Detector Double-Beam Modulation for High-Sensitivity Infrared Spectroscopy

*Seong-Min Kim, Yow-Ren Chang, Young Jong Lee**

Biosystems and Biomaterials Division, National Institute of Standards and Technology, Gaithersburg, MD 20899, USA

Table of Contents:

Figures S1−S3: the fixed-frequency absorbance traces measured with the one-detector single-beam method, the two-detector double-beam method, and one-detector DBM; offset drifts in the absorption spectra; spectral noise of the absorption spectra measured by the DBM method; and the fluctuation of averaged absorbance at fixed wavelength measured with the one-detector single-beam method and the two-detector double-beam method.

**
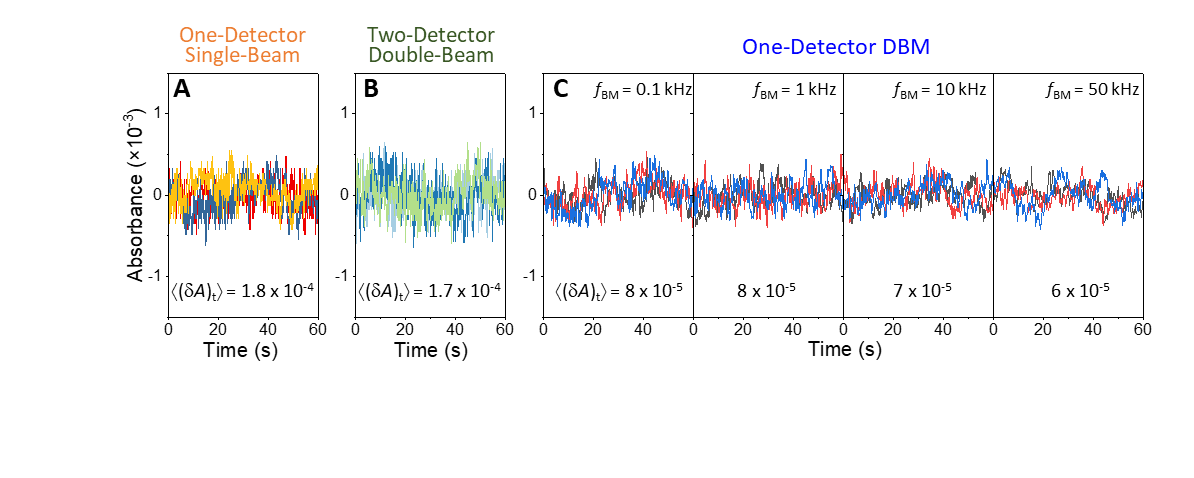
**

**Figure S1.** Fixed-frequency absorbance traces by (A) the one-element single-beam method, (B) the two-element double-beam method and (C) the DBM method. The mean value of each trace was used as the reference value for absorbance calculation. The laser frequency was fixed at 1376 cm^-1^. The LIA time constants were 10 ms, and the sampling time was 0.1 s. The standard deviation of each trace was used to represent the temporal fluctuation, denoted as (δ*A*)_t_, and 〈(δ*A*)_t_〉 indicates the mean of three (δ*A*)_t_ values.


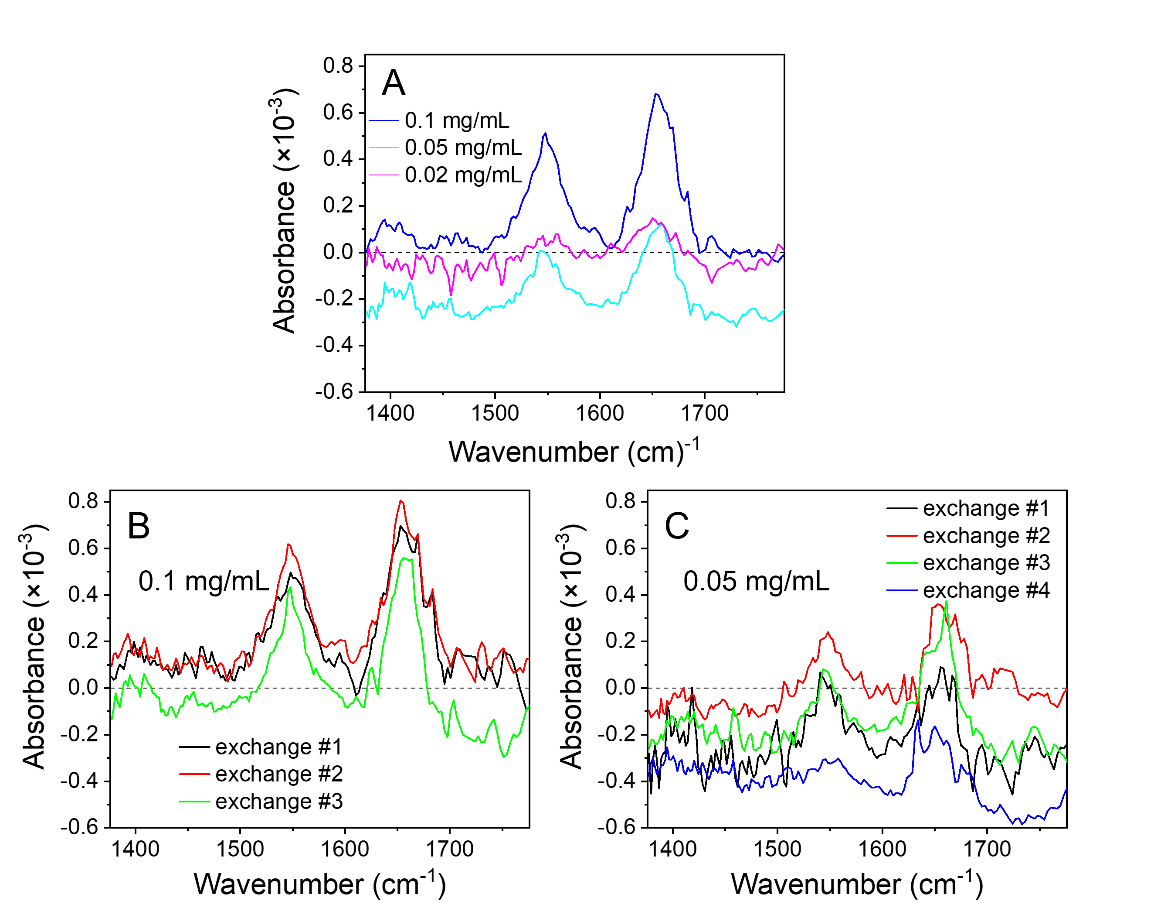


**Figure S2.** (A) Original averaged absorption spectra of BSA solutions before offset drift was subtracted for the spectra in Figure 4D. (B,C) Absorption spectra measured every single cycle of solution-solvent exchange. The time for each solution-solvent exchange cycle was 36 min. The frequency-averaged standard deviations of vertical drifts were (B) 0.12 and (C) 0.18.


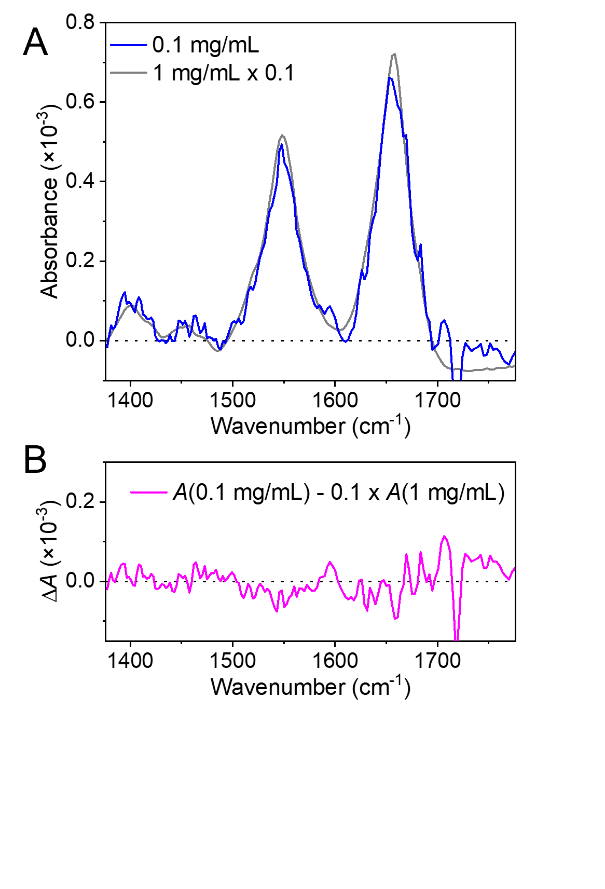


**Figure S3.** Spectral noise of the absorption spectra in Figure 4C,D using the high-concentration and low-concentration BSA spectra. The standard deviation of the absorbance data in (B) is 0.040. It took 36 min for three cycles of sample/reference exchange for each solution.
